# Xylooligosaccharide Production From Lignocellulosic Biomass and Their Health Benefits as Prebiotics

**DOI:** 10.1155/2024/6179375

**Published:** 2024-11-05

**Authors:** Kajal Kumari, Sushil Nagar, Sakshi Goyal, Sonu Maan, Vishal Chugh, Vinod Kumar, Neeraj Kharor

**Affiliations:** ^1^Department of Biochemistry, College of Basic Sciences and Humanities, CCS Haryana Agricultural University, Hisar 125004, Haryana, India; ^2^Department of Basic and Social Sciences, College of Horticulture, Banda University of Agriculture and Technology, Banda 210001, Uttar Pradesh, India; ^3^Division of Biochemistry, Faculty of Basic Sciences, Sher-e-Kashmir University of Agricultural Sciences and Technology of Jammu, Main Campus, Chatha, Jammu 180009, India; ^4^Forage Section, Department of Genetics and Plant Breeding, College of Agriculture, CCS Haryana Agricultural University, Hisar 125004, Haryana, India

**Keywords:** health benefits, lignocellulosic biomass, prebiotic, probiotic, xylanase, xylooligosaccharides

## Abstract

Lignocellulosic biomass (LCB) comprising of wheat bran, coconut husk, rice husk, cereals straw, and other hardwood and softwoods is a good source for the production of xylooligosaccharides (XOS) (prebiotic). XOS produced are nondigestible carbohydrates being stable under stomach pH and digestive enzymes so they can be easily delivered to the intestine in native form, thus stimulating the growth of probiotics. Here we review about the raw material, production, purification, and application of XOS with health benefits. Importance of XOS being valuable food ingredient is increasing as they perform a variety of functions, including reduction in cholesterol levels, gastrointestinal health maintenance, anticancer and antioxidant properties, and modulation of immune system. We also discuss the different characterization methods which are necessary to determine the degree of polymerization (DP) of XOS. Low DP (xylobiose and xylotriose) is usually preferred for the application of XOS in various sectors. This review emphasizes the growing significance of XOS as a prebiotic, serving as nourishment for probiotics.

## 1. Introduction

The Food and Agriculture Organization (FAO) recently estimated that 12.5% of the world's population is suffering from undernourishment. Malnutrition can lead to difficulties in the future, such as the emergence of many diseases, especially noncommunicable diseases such as hypertension and poor digestion, which are considered a threat to adults [1]. Therefore, international attention is focused on new functional foods to meet age problems and other medical needs. The value addition plays an important role as it can be defined as a process during which primary product leads to a high price product by upgrading quality, processing, and other such things. Agriculture has enabled us to achieve food security, but it needs some more ideas in terms of nutrition security. One of the best strategies to make foods digestible and functional is to enrich them with prebiotics. Nondigestible oligosaccharides (NDOs) or prebiotics contain different oligosaccharides and polysaccharides that are resistant to human digestive enzymes and help the host by controlling the growth of the gut microbiota [2]. Lignocellulosic material (LCM) represents the most abundant waste globally [3, 4]. Utilizing these residues for xylooligosaccharides (XOS) production not only adds value but also promotes sustainable development and boosts the economy. Along with production of XOS, wheat bran being an agriculture residue could also be used for production of enzymes like xylanase [5] which further contributes in many other applications including juice clarification [6]. Among the listed oligosaccharides, XOS can be considered as a promising candidate. The XOS are emerging prebiotic oligosaccharides containing xylose residues with β-1,4 linkage between them. Xylose residues numbered 2 to 10 are classified as XOS (xylooligosaccharides), namely, xylobiose, xylotriose, xylotetrose, xylopentose, and further [7]. XOS can be generated from different kinds of raw materials such as hardwoods and various other softwoods comprising of corn straw, corn cob, cereal straws, bagasse, cassava peel, and different types of brans. As an emerging novel source of prebiotics, XOS can be supplemented in food in the form of functional ingredients [8]. Interest in field of XOS patent is increasing as per their numerous industrial applications.

XOS exhibit prebiotic effect to sustain balanced growth of beneficial intestinal microflora, bifidobacteria and lactobacilli [9]. XOS with a good prebiotic effect seem to exert many nutritional benefits and are thus considered as a possible ingredient in functional food. Considering the health benefits, the upcoming challenge is to provide XOS at economic price, particularly in developing countries [10]. For food application, XOS with low degree of polymerization (DP) are considered better and effective [11]. A number of food saccharides (oligo and polysaccharides) as well as the dietary fiber are proposed to have benefits like prebiotic but it is not true in case of all dietary carbohydrates to be a prebiotic [12]. Oligosaccharide-based prebiotics have gained significant attention over time due to their beneficial effects on human health and livestock. Apart from provoking growth of beneficial gut microflora, XOS consumption also led to reduction of cholesterol levels, blood glucose, and procarcinogenic enzymes, along with stimulated immune activity and mineral absorption [13]. Considering the antibiotics ban (as a feed supplement in livestock), XOS could also be used as a future alternative to guard or protect gastrointestinal tract from pathogenic microorganisms [14]. This review stands out from other recently published reviews on the same topic due to its comprehensive analysis of the subject matter. This review hints upon various aspects superficially for the general understanding about the research of XOS using plant biomass and also a summarization of existing literature.

### 1.1. Prebiotics: The Food for Probiotics

A prebiotic is defined as NDOs, being very stable to low pH that can be delivered without being hydrolyzed to the intestine. According to FAO, prebiotic is considered as nonviable food ingredient which assures health benefit associated with modulation of microbiota in the host [14]. More clarified statement to be a prebiotic is being a specifically fermented ingredient proceeding toward particular changes, in framework and activity of gut microflora in order to achieve benefits [15]. Prebiotics like XOS can act as a carbon source for probiotics, providing nourishment that enhances their activity [16]. The most studied prebiotics are XOS, inulin, fructooligosaccharides (FOS), and galactooligosaccharides (GOS), both in human and aquaculture [17]. Prebiotics or the NDOs are naturally present in milk, honey, fruits, and other vegetables like garlic, artichoke, lentils, and barley [18]. Prebiotic concentrations in these natural sources range from 0.3% to 6% of fresh weight. Some general prebiotics are enumerated in Table 1. For any NDOs to be specified as a prebiotic, it must be able to come under the following characteristics firstly not to be hydrolyzed in upper portion of digestive tract. Secondly, they should have the capacity to be specifically fermented by a selected number of beneficial gut microbiota (*Bifidobacterium* and *Lactobacillus*), and lastly they must be able to contribute toward health benefits.

### 1.2. Probiotics: The Consumers of Prebiotics

Probiotics are gut microflora that live in the intestines of humans and animals, contributing to digestive health. The FAO defines probiotics as live microorganisms that, when present in adequate and balanced amounts, provide health benefits to the host [15]. Generally, the most studied probiotics are belonging to species of *Bifidobacterium* and *Lactobacillus*. Consuming probiotic-rich foods may replenish the beneficial gut microflora, for the purpose of gastrointestinal health or prevention of diseases [16, 24]. The stability of XOS and FOS in the presence of intestinal enzymes was studied and confirmed through digestibility test with different probiotics and *S. typhimurium* [25]. Several recent research works have targeted the consumption of XOS by bifidobacteria and lactobacilli; almost all species of *Bifidobacterium* are capable to use XOS [26]. Short-chain fatty acids (SCFAs) are produced through the prebiotic fermentation by gut microflora in the form of acetic acid, propionate, butyric acid, and lactate.

These SCFAs during anaerobic respiration behave like electron sink and also help in mineral bioavailability, reducing gut infection and colon cancer, thus bringing a wholesome improvement in gastrointestinal health [27].

## 2. Chemical Structure of XOS and Xylan

**Figure 1:**
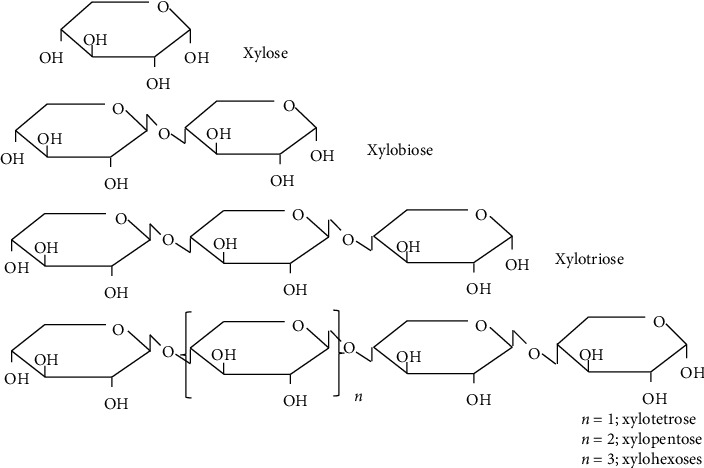
Schematic structure of xylose and xylooligosaccharides.

XOS commercialized in white powder form are made up of two to ten xylose units with β 1–4 linkage (Figure 1); moreover, the molecules with ≤ 20 DP also come under category of XOS [28]. XOS are composed of different types of side chains such as acetyl, methyl, and arabinofuranosyl on the basis of the raw materials and production methods [29]. Structural properties of the XOS were determined using techniques like Fourier-transform infrared spectroscopy (FTIR) and nuclear magnetic resonance (NMR). XOS stabilities vary on the basis of oligosaccharides type, linkage, anomeric configuration, and ring structure formations. Oligosaccharides typically undergo hydrolytic degradation under conditions such as acidic pH (4.0), short-term exposure to elevated temperatures, and prolonged storage. These conditions can alter their nutritional properties and induce various physicochemical changes. XOS show stability with a broad pH range of 2.5–8.0, also at the acidic pH of gastrointestinal juices along with temperatures ranging up to 100°C, thus taking account for XOS preference over the other prebiotics [28].

Xylan is a homopolymer composed of β-(1,4)-D-xylose residues with various types of side linkages including glucuronic acid, 3-O-methyl-glucuronic, α-L-arabinosyl, and α-D-glucuronyl units [30]. Depending on substituent nature, the xylan can be grouped into following four types: (1) arabinoxylans, with side chains of α-L-arabinofuranosyl units using α-(1 ⟶ 3) and/or α-(1 ⟶ 2) linkages; (2) glucuronoxylans, possessing derivatization of α-D-glucuronic acid incorporated with 4-O-methyl ether linked by α-(l ⟶ 2) linkages; (3) glucuronoarabinoxylans, comprising of α-D-glucuronic acid/α-L-arabinose with ester and α-(l ⟶ 2) linkages; and (4) galactoglucuronoarabinoxylans, substituted with β-D-galactopyranosyl residues [31].

### 2.1. Raw Materials for XOS Production

A large amount of LCB is produced from food industry and other sectors. LCB mainly consists of three components i.e., hemicellulose, cellulose, and lignin (Table 2), and the percentage of each component varies on the basis of the type of biomass [42].

The range of all the three LCB components is between 30%– 50%, 20%–40%, and 15%–25% of cellulose, hemicellulose, and lignin, respectively, on the basis of weight/weight [43]. Various LCMs including rice bran, coconut husk, rice husk, cereal straw, and other hardwoods and softwoods are good sources for the production of XOS. Recent research is on the production of XOS from various biomasses such as banana stems, pineapple peels, brewery waste, olive peels, and peanuts, among others [44–47]. Sugarcane bagasse, a lignocellulosic by-product generated in significant quantities during sugar and ethanol production, is a prime candidate for producing XOS. In countries like Brazil, where sugarcane is extensively cultivated, each ton of sugarcane produces approximately 30% bagasse. This by-product contains a substantial amount of xylan, making it a valuable and promising source for XOS extraction [4]. With the expansion of global agriculture, wheat straw production is increasing significantly. China alone contributes over 900 million tons of straw annually, predominantly from corn, rice, and wheat [48].

### 2.2. XOS Production Strategies

#### 2.2.1. Pretreatment and Extraction of Xylan

Various pretreatment protocols have been utilized for complete xylan extraction from hemicellulose. LCM is resistant to chemical and biological degradation, i.e., biomass refractory. Many factors, such as the crystal structure of cellulose, the degree of lignification, and the structural heterogeneity and complexity of the cell wall, cause biomass resistivity of lignocellulosic raw materials that must be overcome for the valuable utilization of LCMs [49]. The main objective of pretreatment is to deconstruct inherent recalcitrant constructions in plant biomass. A number of pretreatment strategies have been studied and developed. Pretreatment causes physical, biological, and chemical changes in the structure of biomass; therefore, it is important to consider the type of pretreatment. Due to different cell walls, pretreatment results are also different. Methods of pretreatments are generally categorized into four types: physical, physiochemical, chemical, and biological. The pretreatment setup determines the potency of the process; higher the yield produced of oligosaccharides, better the process [43].

**Figure 2:**
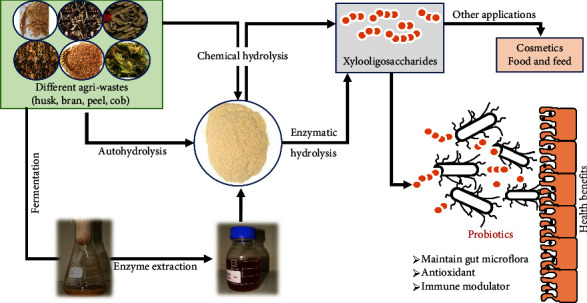
Diagrammatic representation of XOS production strategies and their associated benefits.

Physical treatment methods include grinding, ultrasonic, and microwave treatment, but these use a lot of energy and are often used as auxiliary procedures. Chemical treatment methods have the advantage of high efficiency. However, traditional reagents have disadvantages such as incomplete recovery of polysaccharides, the possibility of producing inhibitors, and the risk of environmental pollution affecting production. In recent years, ionic liquids (ILs) have begun to be used in pretreatment due to their nonflammable, nonvolatile, and recyclable properties. A diagrammatic representation of XOS production strategies along with their benefits is summarized in Figure 2.

#### 2.2.2. Physical Pretreatment

Physical processing for LCB is essential before other treatments. It is used only to reduce the particle size, thereby increasing the surface area and reducing the DP and crystallinity. Autohydrolysis is a physical process that leads to release of acetic acid produced by the cleaving acetyl groups present in xylan chain as ester linkages which are not tolerable to elevated temperatures. This release of acetyl groups is accountable for medium acidification, yield determination, and DP of XOS [21]. The process generated XOS with a broader polymerization range (DP2–DP20) [50]. In autohydrolysis, no catalyst is required; on the other hand, acid hydrolysis takes place with addition of dilute mineral acid making this cost high [51]. Autohydrolysis has one advantage of eliminating corrosive chemicals for extraction but requires equipment which can be operated at higher temperature and pressure [52]. The main disadvantage of physical treatment method is the consumption of high amount of energy. Recent research highlighted that microwave-assisted pretreatment of sugarcane leaves yielded 3.49 mg/mL of XOS, while hydrothermal pretreatment produced 4.95 mg/mL. By combining these physico-biological treatments, the study achieved a total XOS yield of 9.42 mg/mL from sugarcane leaves [53].

#### 2.2.3. Chemical Pretreatment

After the pretreatment using either acid or alkali, the process was further enhanced through enzymatic hydrolysis. After pretreatment, the structural accessibility of LCMs has been enhanced, facilitating easier extraction of xylan. In case of acid hydrolysis, breaking of glycosidic linkage is taking place and the acid reagents are required in low concentration to prevent monomer formation during hemicellulose hydrolysis. In acid hydrolysis, breaking of xylan chain occurs with random attack, thus reducing the DP by releasing monosaccharides [21]. It was reported that acetic acid prehydrolysis of corncob yields 49 mg of XOS per gram of corn cobs at pH 2.7, 150°C after 30 min of incubation [54].

Alkali pretreatment is a well-researched method for chemical pretreatment, focusing on lignin solubilization in alkali solutions. Commonly used alkaline reagents include sodium hydroxide, potassium hydroxide, calcium hydroxide, and ammonium hydroxide, with sodium hydroxide proving most effective. This pretreatment alters the lignocellulosic structure by swelling cellulose, thereby reducing crystallinity and polymerization degree and increasing internal surface area [55, 56]. Additionally, it removes acetyl groups and uronic acid substitutions in hemicelluloses, enhancing accessibility of carbohydrates to enzymatic hydrolysis [48]. Compared with acid hydrolysis, alkaline pretreatment better exposes xylan to hydrolases, thus increasing the XOS yield [28]. Ultrasound-assisted alkali extraction has been shown to notably enhance the XOS yield from corn cobs, with the released amount reaching 174.81 mg/g [57].

ILs are a new class of solvents containing cations and anions with melting points < 100°C. In the first stage, cations and anions play an important role in loosening cellulose and lignin. Most ILs can be recycled and reused. They have important advantages such as low vapor pressure, low energy, nontoxicity, good thermal and chemical stability, and, most importantly, tunable properties of cations and anions on which the function of the IL depends. Due to their unique chemical properties, a significant drawback of ILs is their high cost and their detrimental impact on bacteria and enzymatic action.

#### 2.2.4. Biological Pretreatment/Enzymatic Hydrolysis

**Figure 3:**
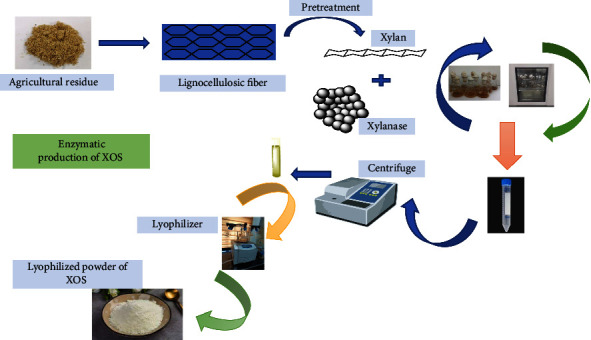
Diagrammatic representation of xylooligosaccharide production through enzymatic hydrolysis.

Biological pretreatment is a low-cost and environmentally friendly technology for treating LCM before enzymatic conversion to sugar. The technology is promising because no reaction residues are produced during operation, and it requires less energy and is environmentally friendly. In this way, lignin-degrading bacteria or fungi are used to pretreat LCM as whole cells or enzymes. Enzymatic method (Figure 3) has several advantages such as their specificity, high reaction rates, and control over the reaction. The enzymatic hydrolysis process is more environment friendly, carried out in moderate conditions, and does not use harmful substances, thus complying with the biodegradable point of view. Also, enzymatic hydrolysis of the hydrothermal liquor has been found to generate higher short-XOS yields as compared to the alkaline pretreatment [58].

Xylan hydrolyzing enzymes have gained attention for their widespread administration in industrial operations such as bio-bleaching of pulp, textile industry, wastewater treatment, production of XOS and juices, and wine clarification [59]. Additionally, the use of xylan-degrading enzymes increases efficiency and selectivity, allowing for low DP XOS and reducing the identification of undesirable products [49]. Hemicellulose possesses a wider range of chemical linkages, so to perform complete hydrolysis, a larger enzyme stock is needed. The required enzymes are endo-β-1,4-xylanases, 1,4-β xylosidase, α-arabinofuranosidase, acetylxylan esterases, feruloyl esterases, etc. [50]. Nagar et al. reported that alkali-stable, cellulose-free xylanase by *B. pumilus* SV-85S over wheat bran in place of xylan shows its effectiveness in paper and pulp production; also, the manufacturing cost of enzyme was reduced to 50% [60]. Production of XOS from cassava peels through NaOH pretreatment and enzymatic hydrolysis yielded 396.5 mg XOS/g xylan. Recovering up to 3.27% XOS from the peels highlights the technical feasibility of utilizing cassava peel residues for generating valuable prebiotic compounds, supporting advancements in sustainable biorefinery and bioeconomy initiatives [61].

Production of XOS by enzymatic means takes places under two steps. First, the hemicellulose from raw material should be extracted through pretreatment methods. During extraction, it should be taken into account that isolated hemicellulose characteristics depend on the pretreatment method. One more parameter that should be considered during the process protocol is the LCM heterogeneity which is required for the isolation of the desired polysaccharide. Second step proceeds with enzymatic degradation of xylan, which is done by the action of β-1,4-xylanase, required character for more production of oligomers with minimum interference of monomers. The endo-β-1,4-xylanase performs and cleaves on xylan backbone to produce low DP XOS. In our previous study, we achieved a XOS yield of 374 mg per gram of wheat bran xylan using a combination of chemical pretreatment followed by enzymatic hydrolysis with *Enterobacter hormaechei* KS1 xylanase. This was achieved under conditions of pH 6.0, a temperature of 50°C, and an incubation time of 24 h [62]. Xylanase activity was also enhanced through enzyme immobilization means on suitable carrier matrix as immobilization increases the stability and availability of the enzyme [63]. A two-way strategy was also studied for production xylanase from wheat bran and again using the residual wheat bran as a support material for xylanase immobilization [64]. For better and efficient hydrolysis of hemicellulose, backbone acting enzymes are necessary. Production of xylanase from different microbial sources is listed in Table 3.

### 2.3. Purification and Separation of XOS

XOS obtained from thermochemical or enzymatic hydrolysis method generally contain a wide DP of oligomers. In order to obtain high purity and desired range of DP, it is necessary to remove monosaccharaide (including xylose) or nonsaccharide compounds. Xylose, along with other monosaccharides such as glucose and arabinose, is counted as an impurity to XOS. High purity of XOS at the analytical scale can be achieved by chromatographic separation technique. The products obtained from hydrothermally treated LCB are fractionated by the use of size -exclusion and anion exchange chromatography. Various techniques like NMR, matrix-assisted laser desorption/ionization-time of flight (MALDI-TOF), and mass spectroscopy (MS) have also been employed in the refining of XOS before their structural characterization [52].

Bio-gel columns with different pore sizes are combined to achieve maximum separation purity. A method was successfully made for the purification of XOS using nanofiltration (NF) membrane from the alkali pretreated hydrolyzate of empty fruit bunch by the use of concentrated endoxylanase from recombinant *Streptomyces lividans* 1326 [74]. It was reported that the enzyme purified and concentrated with 70% saturated ammonium sulfate solution and ultra-filtration, respectively, led to a 3.29-fold increase in xylanase yield. Subsequently, the purified xylanase not only improved the yield of XOS but also enhanced their purity [65]. Several production, purification, and characterization strategies employed for XOS are listed in Table 4. A sequential approach integrating polystyrene divinylbenzene (PS-DVB) resin separation, enzymatic hydrolysis, and fermentation process was assessed. This strategy yielded high-quality XOS production and facilitated the lignin removal. NMR technique was used for the determination of structural characteristics of XOS [75]. XOS obtained from cassava pulp were purified by ultrafiltration method using cellulose membrane with cutoff of 12 kDa [82]. The activated carbon adsorption method, coupled with desorption using an ethanol gradient, is widely utilized in XOS separation and purification processes due to its effectiveness and cost-efficiency. This technique relies on varying adsorption strengths at specific sites to differentiate between sugars and impurities. Active carbon plays a crucial role in purifying target compounds by selectively adsorbing substances such as colorants, proteins, and sugars, with optimization achieved through specialized adsorption columns [83].

### 2.4. Detection, Quantification, and Characterization of XOS

The presence of XOS in the hydrolyzate was determined over thin layer chromatographic (TLC) plates either on glass plate or the silica gel 60 F254 (Merck, Germany) by a number of researchers.

Various solvent systems were reported for TLC analysis such as 2-propanol, ethyl acetate, water, and nitromethane in a ratio (v/v) of 6:1:2: [14, 84], a mixture of (2:1.1:1) n-butanol, acetic acid, and water [74]. According to reports, spots on TLC plates are typically developed by spraying with orcinol reagent at temperatures ranging from 90°C to 120°C. Identification and quantification of each component of XOS mixture can be performed with gas chromatography (GC) or high-performance liquid chromatography (HPLC).

The HPX-87H column is widely used for the measurement of glycosyl composition of XOS as it provides stable and near-baseline resolution of glucose, xylose, and arabinose like biomass sugars [52]. In a recent study, analysis of XOS mixture produced from wheat bran through Dionex UltiMate 3000 HPLC supplied with Kromasil C18 column (150 × 4.6 mm) and a diode array detector (DAD) was performed. Acetonitrile and ammonium acetate buffer (pH 5.5) in composition of 20:80 (v/v) was taken as mobile solvent and results recorded at 245 nm indicated the presence of xylobiose and xylotriose [85]. XOS were also analyzed with an Agilent HPLC connected with refractive index detector, along with mobile solvent containing acetonitrile and water (63:37) at 0.5 mL/min of isocratic flow through ZORBAX carbohydrate column with 30 min run. The XOS produced were quantified by considering the peak areas in comparison with standard XOS mixture; total yield was expressed as mg/mL [84]. Characterization of purified XOS can be performed by using few techniques like high-performance size exclusion chromatography (HPSEC), qualitative sugar analysis by paper chromatography (PC), high-performance anion exchange chromatography (HPAEC), MALDI-TOF-MS, FT-IR, and NMR. Although HPX-87H can identify glucose, xylose, and arabinose, it is tailored for organic acids and has low resolution for oligosaccharides. In that sense, HPAEC also stands out as a technique for XOS identification and quantification because it allows for high-resolution XOS separation [86].

## 3. Applications of XOS

**Figure 4:**
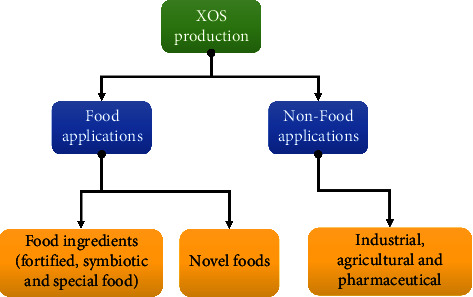
The application of xylooligosaccharides in various fields.

Applications of XOS are in many fields such as in various foods since 1980s (table food, baked food, various candy food, desserts and snacks especially yogurt, fermented, or nonfermented milk, etc.), drug delivery and cosmetics, product for diabetics, agriculture, and pharmaceutical industry (Figure 4).

In pharmaceutical sector, the use of XOS was in antitumor drugs as these are required during preparation of microparticles wilful for delivery of drugs. Extensively, XOS appealed as constituent in functional foods as these are added to soft drinks, confectionery, and other products for children [87]. Due to their stability across a wide range of temperatures and pH levels, along with their prebiotic effects, XOS are increasingly recognized for use in synbiotic foods. Also, the XOS can be used for domestic animals in feed and helpful in agricultural sector in form of yield enhancer and growth stimulator [88].

### 3.1. Biological Benefits of XOS Administration

**Figure 5:**
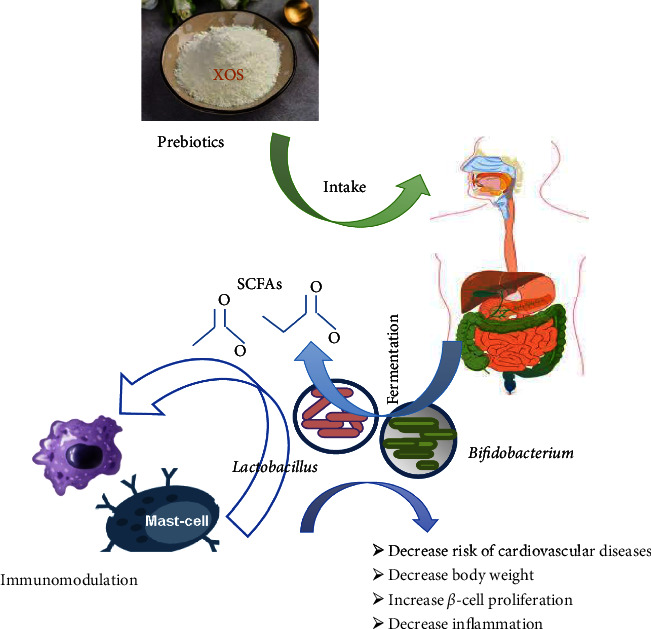
Diagrammatic representation showing biological effects of prebiotics.

Considering their stability, XOS can be additives in various kind of foods and have a number of beneficial biological application as shown in Figure 5 [11].

### 3.2. As Immune Modulators and Immune Stimulators

Beneficial microbes are good to generate antimicrobial agents which promote immune response. Direct impact of prebiotics on immune system is by means of cellular receptor activation and these prebiotics that influence directly are additionally categorized as immunosaccharides or immunostimulants [89]. As an anti-inflammatory factor, XOS (0.1–100 *μ*g/mL) suppress TNF-α (tumor necrosis factor) generation, IL-1β, IL-6, and NO (nitric oxide), while on the other hand, they induce the production of IL-10 in lipopolysaccharide (LPS)-stimulated RAW264.7 cells in dose-dependent manner without inducing cytotoxicity [90, 91]. Prebiotic mediates immune reaction via release of SCFAs and modulating the characteristics and function of gut-associated lymphoid tissues (GALTs). Partially O-acetylated and deacetylated XOS derived from almond shells demonstrate immune-stimulating properties by promoting the proliferation of thymocytes in rats when stimulated with T-cell mitogens. This indicates their potential as compounds that enhance immune function [27].

### 3.3. Anticancer Properties of XOS

Consumption of usable probiotics and prebiotics is correlated through anticarcinogenic properties by stimulating genotoxin detoxification in the gut. The above stated mechanism was studied practically by using carcinogen 1,2-dimethylhydrazine from rat colon and further identifying endpoints which line up tumorigenesis to damage of DNA. XOS have been reported for deterrent effects on cancer and suppressive results on carcinogenesis [91]. MTT (3-(4,5-dimethylthiazol-2-yl)-2,5-diphenyl tetrazolium bromide)–based procedure (colorimetric) was used for identifying XOS anticancer activity. The basic principle for MTT method is inhibition of MTT dye to insoluble crystals which through suspension led to production of purple color. The intensity of purple color production is proportional to viable cell counts and measured by spectroscopy at 570 nm [92]. Hsu et al. studied that XOS and FOS dietary supplementation inhibits the growth of precancerous lesions and promotes the development and activity of bifidobacteria in rats, concluding that XOS were more successful and productive than FOS supplementation [13].

### 3.4. Antioxidant Properties of XOS

It is credited that the continuous use of prebiotics helps to protects against various disease conditions including inflammatory diseases, diabetes, cancer development, abnormal metabolism, and cardiovascular and gestational diseases [40]. Oxidative stress exhibits a precise role in adding up of all these diseased conditions. XOS exhibit antioxidant activity because of the hydroxycinnamic acid derivatives present in them with ester-linkage, namely, ferulic acid, caffeic acid, syringic acid, and coumaric acid, and also due to methyl glucuronic acid ramification on xylan chain. It was also reported that XOS intake with milk and *Lactobacillus* efficiently stimulates antioxidant capacity and the antioxidant potential was measured by DPPH (2,2-diphenyl-1-picrylhydrazyl) and FRAP (ferric reducing antioxidant power) assays [93].

Acidic xylooligomers with uronic acids have shown antioxidant and antiallergic properties [91]. XOS obtained from sugarcane straw by enzymatic hydrolysis exhibit concentration-dependent antioxidant activity up to 78% in a 2 g/L of XOS concentration [40]. Corn cob XOS antioxidant impacts determined by DPPH, 2,2′-Azinobis (3-ethylbenzothiazoline-6-sulfonic acid ammonium salt) (ABTS), FRAP, and oxygen radical absorbance capacity (ORAC). Antioxidant activity was found 49.68 and 64.95 *μ*mol TE/g with FRAP and ORAC while the EC_50_ values by DPPH and ABTS were 1126.00 and 651.69 *μ*g/mL considering the good antioxidant capacity [94]. Enzymatic hydrolysis along with ultrasound irradiation were done for antioxidant XOS production from wheat chaff and antioxidant potential was determined in vitro with ABTS radical scavenging activity assay and it was found 1.03 *μ*mol AAE/g. It was also concluded that antioxidant capacity was largely based on mono and oligosaccharide ratio in XOS [95].

### 3.5. Prebiotic Potential of XOS

Prebiotic efficacy is determined by any selective probiotic growth stimulation. As per literature, *Bifidobacterium lactis* strains exhibited a high specificity for fermenting XOS, while lactobacilli showed a preference for lactitol. In contrast, GOS, FOS, and gentiobiose were utilized by a broader range of microbes [96]. Microorganisms degrade and utilize XOS on the basis of strain specificity and oligomer DP in XOS mixture [6]. XOS resistance to gastrointestinal tract enzyme action was confirmed through in vitro digestibility tests. This in vitro XOS fermentation evaluation showed the best ability of *L. paracasei* and *B. longum* to grow upon these oligosaccharides, specially using xylobiose and xylotriose, thus proving their prebiotic potential [40]. XOS obtained from sugarcane bagasse were tested for hydrolysis in artificial gastric juice, and the degree of hydrolysis decreased as the pH of the juice increased [97]. Furthermore, selective growth analysis has been coupled with SCFA generation measurements, for a more comprehensive assessment of prebiotic activity [98]. Supplementation with 1.0% modified dietary fiber from cassava pulp (M-DFCP) in broiler diets significantly improves gut health. It enhances the populations of beneficial bacteria (*Lactobacillus* and *Bifidobacterium*), increases SCFAs and lactic acid levels, and reduces ammonia production. Therefore, incorporating M-DFCP at 1.0% is recommended for optimizing broiler gut health and performance [99]. Adding prebiotic XOS produced from cassava pulp using *Bacillus* sp. endo-β-1,4-D-xylanase at 5% concentration to MRS medium significantly enhanced the growth of *Lactobacillus acidophilus*, achieving optimal growth and a favorable SCFA profile, indicating the potential of cassava-derived XOS as an effective prebiotic [100].

### 3.6. Human Trial Data on XOS

Among the various increasing prebiotics, XOS gain the most interest because of their usefulness in health, technological, pharmaceutical, and other related properties. Study revealed that XOS incorporation results in changes in composition of intestinal bacterial growth of humans. Additionally, the increase in beneficial microorganisms following the incorporation of XOS has led to a reduction in pathogenic bacteria [16]. It was suggested that XOS consumption at dose of 1.2 to 8 g on daily basis results in beneficial effects on the digestive health [101]. Some of the in vitro fermentation trials and other human trials with varying dose of XOS are mentioned in Table 5.

## 4. Summary and Conclusion

XOS are increasingly recognized as valuable prebiotics due to their ability to positively influence healthy gut microorganisms. They are primarily derived from LCMs via enzymatic processes, which are abundant and cost-effective sources. This sustainable production method allows for increased food output without expanding agricultural land use. Regular consumption of XOS is associated with numerous health benefits, including enhanced digestive health, strengthened immune function, and overall well-being. These properties position XOS as pivotal in the development of functional foods, marking a significant advancement in nutritional science. Future research should focus on determining safe dosage levels of XOS to better understand their disease prevention mechanisms and ensure their optimal utilization in promoting health.

## Figures and Tables

**Table 1:** List of some nondigestible prebiotics.

Type	Abbreviation	Monosaccharide unit	Mode of production	References
Galactooligosaccharides	GOS	Galactose	β-Galactosidase-mediated transgalactosylation	[19]
Glucooligosaccharides	GlcOS	Glucose	Enzymatic synthesis using glycosyl hydrolases and glycosyl transferases	[20]
Xylooligosaccharides	XOS	Xylose	Xylan enzymatic hydrolysis	[21]
Fructoligosaccharides	FOS	Fructose	Inulin hydrolysis and sucrose biotransformation by transfructosylation	[22]
Mannanoligosaccharides	MOS	Mannose	Enzymatic hydrolysis of mannans	[23]

**Table 2:** Composition of raw lignocellulosic materials.

LCMs	Hemicellulose (%)	Cellulose (%)	Lignin (%)	Xylan (%)	References
Corncob	ND	38.50	16–19	36.80	[30]
Hazelnut shell	ND	18.70	46.40	18.70	[32]
Kenaf stem	51.83	37.29	14.38	37.16	[33]
Wheat bran	ND	16–30	5–20	29.00	[34]
Rice husk	28.60	28.60	24.40	10.93	[35]
Coconut husk	26.01	34.12	32.64	24	[36]
Mango seed shell	45.60	18.34	29.42	ND	[37]
Orange fruit waste	10.50	9.21	0.84	ND	[38]
Tobacco stalk	16.99	50.80	15.60	ND	[39]
Sugarcane straw	24–29	38–50	19–25	10.23	[40]
Maize straw	ND	40.52	9.31	34.53	[41]

Abbreviation: ND, not defined.

**Table 3:** Xylanase production and optimized condition for different microbial species.

Microorganism	Substrate	Fermentation	Process parameters	Activity	References
*A. flavus*	Maltose	SmF	37°C, pH 6.5, 96 h	1260 U/mL	[65]
*A. nidulans*	Rice straw	SmF	32.5°C, pH 5, 120 h	11.60 IU/mL	[66]
*A. tamari*	Rice straw	SSF	30°C, pH 5.5, 120 h	180 IU/gds	[67]
*B. amyloliquefaciens*	Cane molasses	SmF	28°C, pH 8.3, 48 h	205 IU/mL	[68]
*B. licheniformis*	Wheat straw	SmF	45°C, pH 9, 24 h	141.28 U/mL	[69]
*B. safensis* XPS7	Sugarcane bagasse	SmF	72 h	10.9 U/mL	[70]
*F. graminearum*	Wheat bran	SmF	29°C, pH 5.79, 154 h	1.79 U/mL	[71]
*T. asperellum*	Palm leaves	SSF	30°C, pH 6	215.42 U/g	[72]
*T. lanuginosus*	Grass leaves	SmF	50°C, pH 7, 72 h	2.7 U/mL	[73]

Abbreviations: SmF, submerged fermentation; SSF, solid state fermentation.

**Table 4:** Different production, purification, and characterization methods for XOS.

Raw material	Method	XOS yield (%)	Purification technique	DP and purity (%)	Characterization	References
Moso bamboo	Autohydrolysis	98.60	Polystyrene divinylbenzene (PS-DVB) resin	X2, X3 (93.20)	NMR	[75]
Bamboo	Enzymatic	7.50	Ultrafiltration, ion exchange	XOS (92.30)	ND	[76]
Sugarcane straw	Autohydrolysis	43.60	Nanofiltration membrane	X2, X3 (20.90)	ND	[77]
*Eucalyptus nitens* wood	Autohydrolysis	57.60	Nanofiltration membrane	X3–X10 (83.15)	MALDI-TOF MS	[78]
Corn straw	Autohydrolysis + enzymatic	96.00	Nanofiltration membrane	X2–X6 (84.15)	ND	[79]
Corn stalk	Enzymatic	40.60	Activated carbon	X2–X8 (96.30)	ND	[80]
Beechwood xylan	Enzymatic	ND	ND	X2–X7	HPLC-RID	[81]

Abbreviation: ND, not defined.

**Table 5:** Prebiotic potential of XOS with different doses based on human trials.

Dose	Participants∗	Disease type	XOS (%)	Results	Samples	Reference
3 g/day	25 (18–80 years)	Colorectal cancer	97.49	• Improved gut microbiota composition • Improved efficacy of chemotherapy	Stool and blood	[102]
1 capsule twice/day	60 (18–65 years)	Irritable bowel syndrome	ND	• Effectively controlled diarrhea • Alleviated clinical symptoms	Stool	[103]
1–3 g/day.	7 adults	Healthy persons	83	• Dose-dependent increase in prebiotic effect	Lumen and dialyzate	[104]
10.4 g/day	30 adults	Overweight	ND	• Boosting of beneficial microbiota • Improved glucose homeostasis	Urine, feces, and blood	[105]
0.1 g/mL	19 years and older	With no gastrointestinal disease	95	• Supports growth of probiotics in vivo	Stool	[106]
0.1 g/mL	3 (23–27 years)	With no gastrointestinal disease	95	• Help to identify specific fibers during irritable bowel syndrome	Fecal	[107]
3 g/day	6 (26–36 years)	Healthy persons	84	• Increased lactate and SCFA levels • Bifidogenic effect	Fecal	[108]
0.5–12 mg/day	6 adults	Healthy adult volunteers	95	• Support the prebiotic activity • Production of SCFA	Fecal	[109]
3.7 g/day	3 (29–35 years)	Healthy adults	ND	• Increased SCFA levels • Helpful to study metabolic disorders	Fecal	[110]

^∗^Participant number and age group.

## Data Availability

The data used to support the findings of this study are included in the article.
